# Mixed Molybdenum Oxides with Superior Performances as an Advanced Anode Material for Lithium-Ion Batteries

**DOI:** 10.1038/srep44697

**Published:** 2017-03-15

**Authors:** Di Wu, Rui Shen, Rong Yang, Wenxu Ji, Meng Jiang, Weiping Ding, Luming Peng

**Affiliations:** 1 Key Laboratory of Mesoscopic Chemistry of MOE and Collaborative Innovation Center of Chemistry for Life Sciences, School of Chemistry and Chemical Engineering, Nanjing University, Nanjing 210093, China; 2 General Motors R&D, Warren, MI 48090, USA

## Abstract

A simple and effective carbon-free strategy is carried out to prepare mixed molybdenum oxides as an advanced anode material for lithium-ion batteries. The new material shows a high specific capacity up to 930.6 mAh·g^−1^, long cycle-life (>200 cycles) and high rate capability. 1D and 2D solid-state NMR, as well as XRD data on lithiated sample (after discharge) show that the material is associated with both insertion/extraction and conversion reaction mechanisms for lithium storage. The well mixed molybdenum oxides at the microscale and the involvement of both mechanisms are considered as the key to the better electrochemical properties. The strategy can be applied to other transition metal oxides to enhance their performance as electrode materials.

As one of the solutions for our new energy-based economy, lithium ion batteries are widely used in mobile devices, such as cellular phones and laptops, because of their relatively high volumetric and gravimetric energy density^1^,^2^. However, in order to meet the requirements for applications in electric vehicles, safe electrode materials with less cost, higher energy density, better rate performance and longer service life need to be designed^3^. Transition-metal oxides, which often have much higher theoretical capacity than traditional graphite materials, have been extensively studied as the anode materials for lithium ion batteries^4^. Molybdenum oxides (including both MoO_2_ and MoO_3_) represent attractive candidates due to their high theoretical capacity (about 838 mAh·g^−1^ for dioxides and 1117 mAh·g^−1^ for trioxides). Bulk molybdenum oxides, however, show much lower capacities compared with their theoretical ones, which can be attributed to the significant volume change during the charge and discharge processes and the intrinsic slow kinetics for the conversion reactions^5^.

Recently, metal oxide nanostructures, especially nanostructured oxides have been prepared as anode materials for lithium ion batteries due to their short diffusion length, larger reaction surface area between the electrode and electrolyte, as well as extra space for better accommodation of the strain induced by large volume changes^6^,^7^,^8^,^9^,^10^,^11^. For example, molybdenum dioxide with a yolk-shell structure achieves a discharge capacity of 847.5 mAh·g^−1^ after 50 cycles at 50 mA·g^−1^ 12. Uniform carbon coated nanospheres exhibit a discharge capacity of 410 mAh·g^−1^ after 30 cycles even at 3 C rate^6^. Coating highly conductive materials, such as carbon, is another strategy to improve the performances of metal oxides by increasing the electrode conductivity, as well as possibly improving the surface chemistry of the active material. The MoO_2_ hollow structures coated with Mo_2_N show a capacity of 815 mAh·g^−1^ after 100 cycles at 100 mA·g^−1^ 13. Nevertheless, it is usually expensive to prepare the complex nanostructure and the capacity fading often occurs during the cycling process, while coating conductive materials with low capacity reduces the specific capacity of the active materials.

Partial oxidation or reduction strategy has been employed to improve the electrochemical properties of cathode materials, for example, controlled reduction of LiV_3_O_8_ generate Li_x_V_2_O_5_/LiV_3_O_8_ composite with significantly improved cycling performancs and rate capability^14^. Herein we report a very simple and convenient method to prepare the mixed molybdenum oxides (MMO, MoO_x_, 2 < x < 3) by moderately oxidizing molybdenum dioxide nanoparticles under mild oxidizing atmosphere. The obtained MMO exhibits a discharge capacity as high as 930.6 mAh·g^−1^ at a current density of 200 mA·g^−1^ after 200 cycles as an advanced material, indicating this material has potential for applications in lithium-ion batteries.

## Experimental Section

### Preparation of the MoO_2_ nanoparticles

MoO_2_ nanoparticles were prepared by using a hydrothermal method reported by Liu *et al*.^15^. In a typical experiment, 1 g of ammonium heptamolybdate tetrahydrate was dissolved into the mixed solvent of 45 mL distilled water and 5 mL ethylene glycol (EG). After that, the mixture was transferred into a 100 mL Teflon-lined stainless-steel autoclave and was kept in an oven at a temperature of 180 °C for 48 h. The resulted black precipitate was centrifuged, and then washed by distilled water and followed by ethanol for three times. The product was finally dried at the 80 °C.

### Preparation of the MMO and MoO_3_ nanoparticles

To prepare MMO, the as prepared MoO_2_ nanostructured powders was heated in a tube furnace at a rate of 5 °C·min^−1^ to 500 °C and kept at the temperature for 100 min. A mixed gas containing a small fraction of oxygen in nitrogen was flowed into the system during heating at a rate of 20 mL/min. The fractions of oxygen were controlled as 0.2% and 1%, in order to achieve the final product MMO and MoO_3_, respectively.

### Materials Characterization

The XRD patterns were acquired with an X-ray diffractometer (Philips Analytical X’pert Pro) at a 2θ range of 10–70°. The diffractometer was equipped with a Cu Kα radiation (λ = 1.5418 Å) and operated at 40 kV and 40 mA. The air sensitive samples were protected by Kapton films in the XRD measurement. The morphology of the samples were investigated using a field-emission scanning electron microscope (Hitachi FE-SEM S4800). X-ray photoelectron spectroscopy (XPS) was used to survey the surface compositions and the chemical states of the samples (PHI5000 VersaProbe, PerkinElmer). The hydrocarbon C 1 s peak at 284.5 eV was chosen as the reference for the binding energies. The XPS spectra were analyzed with the software XPSPeak 4.1. The ratio of the peak area of the Mo 3d_5/2_ peak to the Mo 3d_3/2_ peak is fixed to 1.5 while the binding energy separation is set to 3.0 eV. A Bruker Advance III 400 MHz spectrometer with a 3.2 mm Magic Angle Spinning (MAS) probe was used for acquiring the ^7^Li solid-state NMR spectra at a resonant frequency of 155.46 MHz. A spin-echo and a 2D exchanged pulse sequence were used in the data acquisition. In order to select reduce the intensity of the resonance due to the SEI, a T_2_ filter was used in the spin-echo NMR data acquisition. After discharging to 5 × 10^−3^ V, the self-supported electrode^16^ was taken out, rinsed with dimethyl carbonate (DMC) to remove the residual electrolyte, before it was dried and packed into NMR rotors in an Ar glove box, and spun at 18 or 20 kHz for NMR data collection. A 1 M LiCl aqueous solution was used to set the spectral reference to 0 ppm.

### Electrochemical Characterization

Electrochemical measurements were performed on coin cells (model 2032) with lithium metal foils as the reference and counter electrodes at ambient temperature. 70 wt% of as prepared samples, 20 wt% acetylene black (conducting additive), and 10 wt% polyvinylidene fluoride (binder) were blended with N-Methyl-2-pyrrolidone (NMP) to make a slurry and then spread onto a copper foil current collector. After drying at 80 °C for 6 hours, the electrode was punched and then dried under vacuum at 120 °C for 12 hours. The electrodes were then transferred to an argon-filled glove box where both the moisture and oxygen concentrations were kept below 1 ppm. The electrolyte was prepared by dissolving 1 M LiPF_6_ in a mixture of ethyl methyl carbonate (EMC), ethylene carbonate (EC) and dimethyl carbonate (DMC) (1:1:1 in weight) solution while a Celgard 2325 film was used as the separator. An electrochemical station (CHI660D) was used to obtain cyclic voltammetry (CV) at ambient temperature. CV measurements were performed over a potential range of 5 × 10^−3^–3.0 V (vs. Li^+^/Li) at a scan rate of 2 × 10^−4^ V s^−1^. Galvanostatic discharge–charge measurements were performed at different rate on a battery testing system (LAND CT2001A model, Wuhan Jinnuo Electronics Co. Ltd) with a voltage window range from 5 × 10^−3^ to 3.0 V (vs. Li^+^/Li).

## Results and Discussion

**Figure 1:**
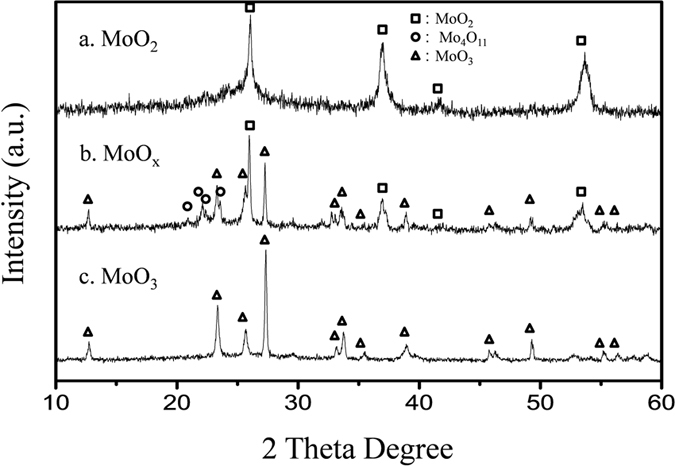
XRD patterns of the MoO_2_ nanoparticles annealed in N_2_ flow with different O_2_ content ((**a**), 0%; (**b**), 0.2%; (**c**), 1% O_2_ in N_2_).

The XRD patterns of different molybdenum oxide nanoparticle samples are shown in Fig. 1. All of the XRD peaks for MoO_2_ nanoparticles annealed with pure N_2_ can be indexed to MoO_2_ (Fig. 1a, ICSD:080830), while the sample heated with a high concentration of O_2_ (1% O_2_ in N_2_) in the flow is completely oxidized to MoO_3_ (Fig. 1c, ICSD:076365)^17^. However, MoO_2_ nanoparticles treated with a smaller fraction of O_2_ (0.2% O_2_ in N_2_) show mixed phases which can be attributed to MoO_2_, MoO_3_, as well as a small fraction of Mo_4_O_11_ (Fig. 1b, ICSD:089340), indicating the nanoparticles have been partially oxidized, noted as MMO. By using the Scherrer equation, the mean diameters of MoO_2_ and MoO_3_ nanocrystallites can be determined as 5–8 and 50 nm, respectively, while the average size of MMO is in between.

**Figure 2:**
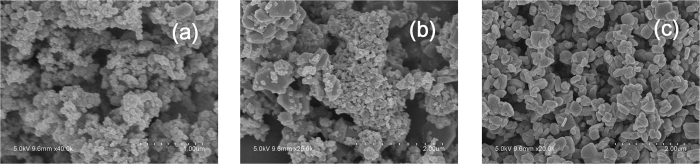
SEM images of MoO_2_ (**a**), MMO (**b**) and MoO_3_ (**c**).

Figure 2 shows the SEM images of the obtained MoO_2_, MMO and MoO_3_ samples. Small agglomerates with a diameter of about 50 nm composed of smaller and round shaped nanoparticles can be observed for the MoO_2_ sample, while MoO_3_ are much larger in size (100 to 500 nm). The SEM image of MMO shows this sample is composed of small particles with different sizes, resembling a mixture of the above two materials. The results indicate that MMO are not uniformly oxidized, in agreement with the XRD data.

**Figure 3:**
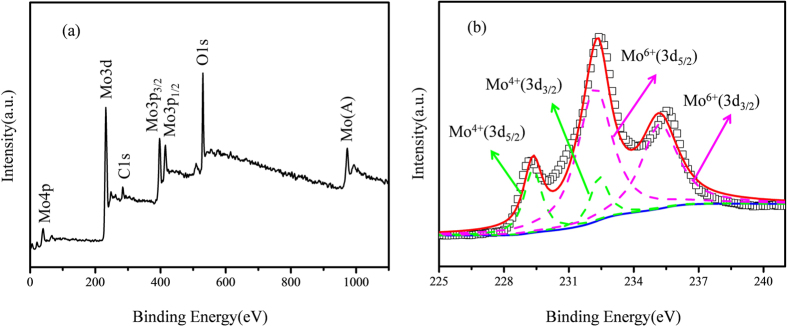
XPS patterns of MMO: (**a**) survey spectrum and (**b**) high-resolution Mo 3d region.

X-ray photoelectron spectroscopy (XPS) measurement was performed in order to investigate the surface valence states of Mo in MMO, and the results are shown in Fig. 3. The peaks of Mo 3d and O 1 s can be observed in the survey spectra (Fig. 3a), and the former was further examined. The two peaks at about 232.0 and 235.0 eV are characteristic of Mo^6+^, and can be attributed to Mo^6+^ (3d_5/2_) and Mo^6+^ (3d_3/2_), respectively^18^. The lower energy peak centered at 229.3 eV can be attributed to Mo^4+^ (3d_5/2_), suggesting that the Mo ions on the surface of MMO are in mixed valence states^19^. The spectrum was fitted into two doublets ascribed to Mo^6+^ (3d_5/2_)/Mo^6+^ (3d_3/2_) and Mo^4+^ (3d_5/2_)/Mo^4+^(3d_3/2_) (Table 1). The Mo^6+^/Mo^4+^ ratio is determined to be 4.0 according to the line fitting results, hence the valence state of molybdenum on the surface of MMO can be determined as 5.6^18^,^20^.

**Figure 4:**
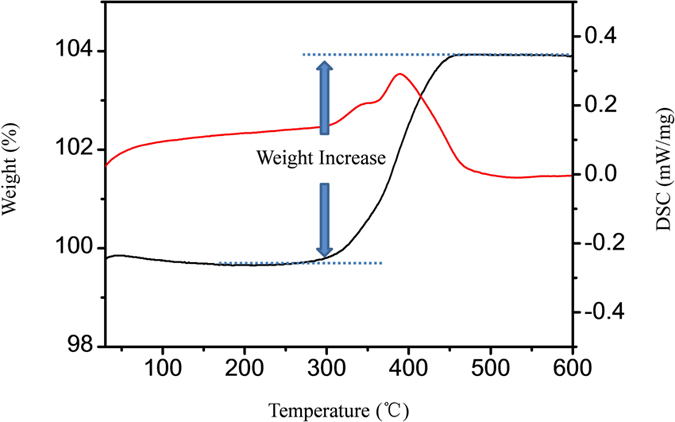
TGA-DSC curve of MMO in air at a heating rate of 5 °C/min.

To further investigate the overall valence state of the MMO, TGA-DSC test was carried out to monitor the weight changes as a function of temperature in the air atmosphere and the results are shown in Fig. 4. The small weight loss (about 0.3%) at the temperature lower than 150 °C can be attributed to the loss of absorbed water. The weight increase at 300–500 °C corresponds to the oxidization of MMO (MoO_x_) to MoO_3_ in the air and a wide exothermic peak is associated with this process. The total weight increase is 4.30% (Fig. 4), and thus x in MoO_x_ can be extracted as 2.63, leading to a calculated average valence state of 5.26 for all Mo ions in the sample MMO. This value determined by TGA is apparently lower than the valence state of surface Mo ions determined by XPS, indicating that the surface of MMO was oxidized to a higher extent compared to the inner part of the material. On the basis of the nominal formula of MoO_2.63_ the theoretical capacity of MMO is 1021 mAh·g^−1^ based on the conversion mechanism.

**Figure 5:**
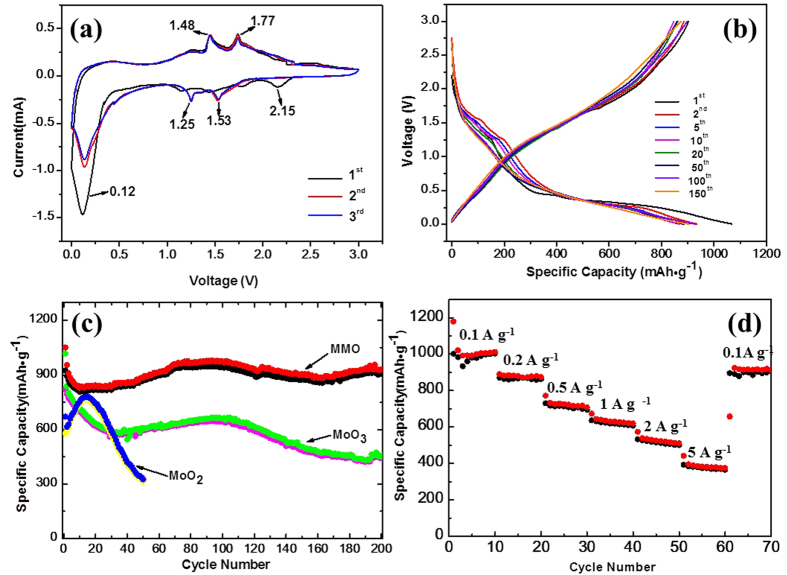
(**a**) Cyclic voltammograms of MMO at a scan rate of 0.2 mV·s^−1^ with a mass of 2.5 mg. (**b**) Discharge and charge curves of MMO at a current of 100 mA·g^−1^ in the potential window of 3–5 × 10^−3^ V vs. Li^+^/Li. (**c**) The specific discharge/charge capacity vs cycle number plots for different samples at a current of 200 mA·g^−1^. (**d**) Specific capacity vs cycle number curves at different current densities.

Cyclic voltammetry (CV) was used to study the Li insertion/extraction behaviors in different samples and the results are presented in Fig. 5a. The redox peaks centered at 1.25/1.53 V and 1.48/1.77 V are attributed to the reversible monoclinic–orthorhombic–monoclinic phase transitions in the partially lithiated Li_x_MoO_2_ according to the CV data of MoO_2_ nanoparticles (Figure s1a) and previous reports^6^,^19^,^21^, consistent with the presence of MoO_2_ in MMO which was illustrated in the XRD pattern and the TG curve^6^,^21^. Besides these two redox pairs, the whole CV curve of MMO is similar to that of our MoO_3_ nanoparticles (Figure s1b) and literature^22^,^23^,^24^ due to the high concentration of MoO_3_, as implied by XRD and XPS data^22^. There are also intense peaks below 0.5 V, which can be associated with a conversion reaction with lithium, where Mo oxides are reduced completely to metallic Mo along with the formation of Li_2_O. The curve changes very little in the following cycles, suggesting that the partially lithiated Li_x_MoO_2_ structure is very stable during the charge-discharge progress, thus MMO is expected to have a good cycling performance^25^. Meanwhile the difference between the first and following cycles can be attributed to irreversible reactions such as the irreversible phase transition, the decomposition of the electrolyte and the formation of a solid electrolyte interface (SEI) layer^22^,^26^.

To further investigate the electrochemical performance of the samples, galvanostatic experiments were performed on the coin cells. Figure 5b shows the charge/discharge plots of MMO at a current of 200 mA·g^−1^. The initial discharge capacity is 1068.2 mAh·g^−1^, slightly higher than the theoretical capacity of MMO (1021 mAh·g^−1^), which can be assigned to some irreversible reactions, e.g. the generation of an SEI layer^23^,^27^. In agreement with the CV results, two short discharge plateaus at about 1.55 and 1.26 V can be observed^13^,^21^. The expected charge plateaus at around 1.4 and 1.7 V are not clearly visible, which is similar to previously reported MoO_3_ nanostructures^26^. The charge/discharge curves are very stable with continued cycling, suggesting good cycling performances, except that the voltage of discharge plateaus decreases and eventually the plateau can no longer be observed. Since the plateaus are usually attributed to the phase transition in the partially lithiated Li_x_MoO_2_, the decrease in voltage can be ascribed to the transformation of crystalline phase to amorphous-like structure^13^,^21^.

As shown in Fig. 5c, the electrochemical performance of MMO is much better than MoO_2_ or MoO_3_. For MoO_2_, the first discharge capacity is only 670.5 mAh·g^−1^ while the capacity gradually increases upon cycling and attains 781.7 mAh·g^−1^ after 15 cycles. This activation process has been reported by other groups and may be attributed to the partial loss of crystallinity of the material during the cycling^13^,^28^,^29^, accompanied with a larger fraction of the material undergoing further conversion reaction instead of stopping at Li_x_MoO_2_ phase^30^. Nonetheless, a rapid capacity deterioration decay can be observed once the capacity reaches the maximum value and only a capacity of 325.7 mAh·g^−1^ remains after 50 cycles. The initial capacity of MoO_3_ is 1017.6 mAh·g^−1^, however, it drops to 593.4 mAh·g^−1^ after only 30 cycles due to the big volume changes during cycling. Such decrease can also be observed in MMO, which has a large fraction of MoO_3_. However, the drop in capacity is much less. The capacity decreases from the first discharge capacity of 1068.2 mAh·g^−1^ to 842.3 mAh·g^−1^ after 20 cycles, corresponding to a capacity loss of 12% (calculated based on the capacity of 2^nd^ and 20^th^ cycles). After that, the capacity increases slowly and reaches 976.3 mAh·g^−1^ after 90 cycles. The discharge capacity of the MMO only decreases slightly to 930.6 mAh·g^−1^ after 200 cycles, which is equivalent to a capacity retention of 95% of the maximum value of 977 mAh·g^−1^, demonstrating high capacity and excellent stability. Such high reversible capacity of more than 930.6 mAh·g^−1^ at 200 mA·g^−1^ is almost three-fold that of commercial graphite (~330 mAh·g^−1^). Although the overall profile of MMO’s cycling curve is similar to that of MoO_3_, the key difference is the capacity decay is much smaller for MMO than MoO_3_. This decay, which can be seen in many reports of MoO_3_, is generally assigned to the huge volume and/or structure change during the conversion reaction^23^,^27^. Since the volume and structure changes are very limited in the insertion reaction^31^, the presence of stable partially lithiated Li_x_MoO_2_ in MMO is expected to help stabilize the whole electrode and avoid the capacity loss. To further investigate the hypothesis above, the electrochemical performance of the mixed MoO_2_ and MoO_3_ sample was prepared by ball milling (MMM) (Figure S2b). Although MMM is also composed of MoO_2_ and MoO_3_ with similar proportion (Figure S2a), the electrochemical performance is apparently worse than that of MMO. Although an initial capacity of 1050 mAh·g^−1^ can be obtained, which is similar to that of MMO, only a capacity of approximately 100 mAh·g^−1^ can retain after 100 cycles at a current rate of 200 mA·g^−1^. The very rapid decay can be ascribed to the phase isolation which is expected to accelerate the pulverization process. Owing to the poor electrochemical performances of Mo_4_O_11_^32^, which is the intermediate phase in the transformation from MoO_2_ to MoO_3_^33^, the small fraction of Mo_4_O_11_ in MMO is also unlikely to be associated with the improved electrochemical properties.

The rate capability of MMO was further investigated and the results are shown in Fig. 5d. The material was tested with the potential range of 5 × 10^−3^–3.0 V at 0.1 A·g^−1^, 0.2 A·g^−1^, 0.5 A·g^−1^, 1 A·g^−1^, 2 A·g^−1^, 5 A·g^−1^ and 0.1 A·g^−1^ for 10 cycles each. The specific capacity reaches 994 mAh·g^−1^ at 0.1 A·g^−1^, 883 mAh·g^−1^ at 0.2 A·g^−1^, 735 mAh·g^−1^ at 0.5 A·g^−1^, 644 mAh·g^−1^ at 1 A·g^−1^, 538 mAh·g^−1^ at 2 A·g^−1^, 397 mAh·g^−1^ at 5 A·g^−1^, respectively. A specific capacity of 397 mAh·g^−1^ at 5 A·g^−1^ for this material is as high as 40% of its capacity at 0.1 A·g^−1^ (994 mAh·g^−1^). The results show that MMO has a better rate capability compared to previous reports^26^,^34^ It is noteworthy that this high performance is achieved without coating the active material with carbon based conductors such as graphene.

Table 2 summarizes some recent works on molybdenum oxide based materials as anodes for lithium ion batteries. The specific capacity (930.6 mAh g^−1^, 0.2 A·g^−1^) and cycling performance of MMO in this work excels most of the molybdenum oxide based materials previously reported in the literature, and even those with carbon coating^35^,^36^,^37^,^38^,^39^,^40^,^41^.

**Figure 6:**
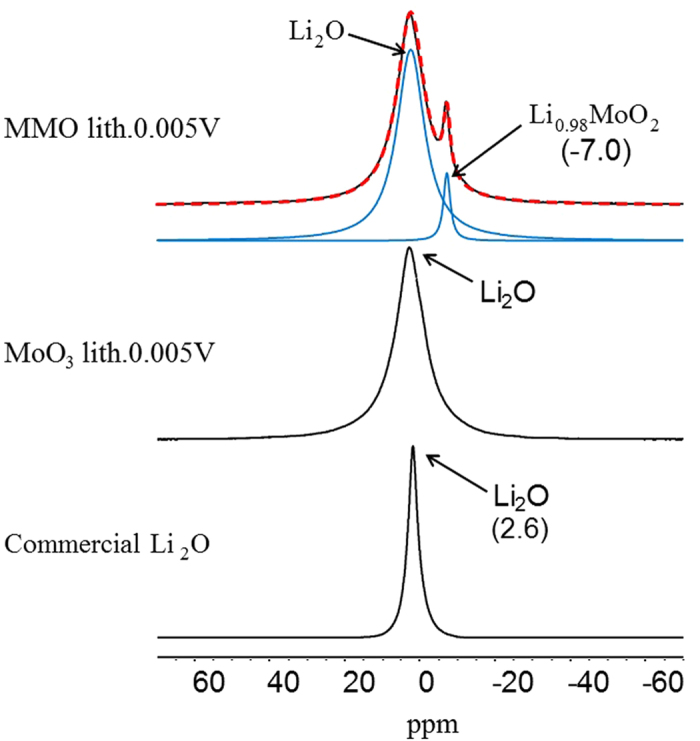
^7^Li solid-state NMR spectra of MMO lithiated to 5 × 10^−3^ V with a simulation (dashed line in red) fitted with each individual component (solid lines in blue), in comparison to MoO3 lithiated to 5 × 10^−3^ V and commercial Li2O, for comparison. Spinning rate: 20 kHz.

To further understand lithium storage mechanisms of MMO, Solid-State NMR and XRD experiments on lithiated samples were performed. ^7^Li NMR spectroscopy proves to be a powerful method to investigate the relationship between local structures and electrochemical performances of materials for lithium ion batteries and to reveal the electrochemical reaction mechanisms^42^. ^7^Li MAS NMR spectra of the MMO and MoO_3_ lithiated to 5 × 10^−3^ V are given in Fig. 6. A broad resonance can be seen at 2.6 ppm in the spectrum of lithiated MoO_3_, which can be attributed to Li_2_O which is the product of the conversion reaction. The resonance of the lithiated oxide is broader compared to the spectrum of commercial Li_2_O standard sample, indicating more complex local structures of the lithium ions after the lithiation process, and/or the magnetic fields generated by the Mo particles^22^. Similarly, the broad peak at 2.6 ppm in the spectrum of lithiated MMO can be assigned to Li_2_O formed in the conversion reaction. A sharp peak at −7.0 ppm can also be observed for MMO, indicating that there is another local environment of lithium ions.

**Figure 7:**
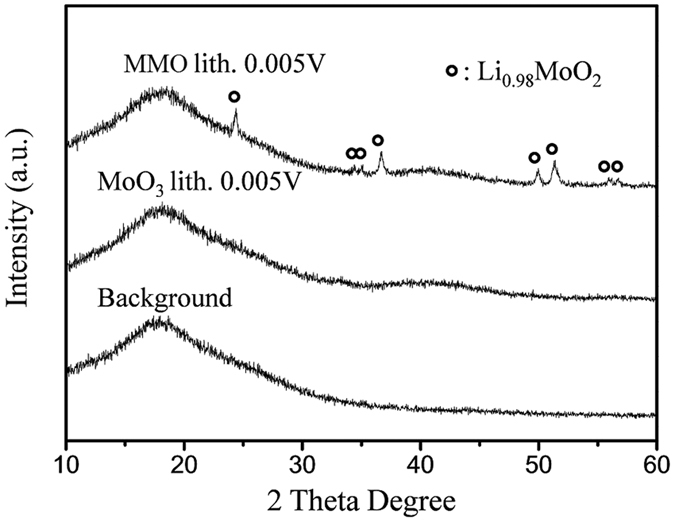
XRD patterns of MMO and MoO_3_ after lithiated to 5 × 10^−3^ V with the protection of Kapton film, in comparison with the background.

The presence of the other lithium compound is further supported by XRD data of lithiated MMO (Fig. 7). The Kapton film was used to protect the air-sensitive fully lithiated sample and its broad background peak can be found between 2θ of 10–30°. No distinct peaks can be seen in the pattern of lithiated MoO_3_, suggesting the poor crystallinity of the product after lithiation. However, pronounced peaks due to Li_0.98_MoO_2_, which was previously reported as the product of the insertion reaction of MoO_2_^30^, appear in the pattern of lithiated MMO. In combination with the results of ^7^Li MAS NMR spectra of MMO after lithiation, it is clear that two mechanisms including both conversion and insertion reactions are involved in the lithiation of MMO (MoOx) while only conversion reaction is associated with MoO_3_. Assuming MoO_3_ in MMO only undergoes a conversion reaction while MoO_2_ is associated with both insertion/extraction and conversion reaction, the fraction of MoO_2_ (*x*) which underwent insertion/extraction reaction in MMO can be estimated in the following equation (considering the theoretical capacity of MoO_2_ with insertion/extraction mechanism is 210 mAh/g):


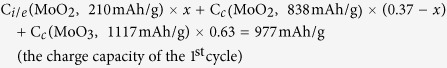


Thus, the value of *x* can be determined as 0.058, implying 16% of the MoO_2_ material underwent the insertion/extraction reaction in the MMO material. The capacity of MMO increased after 50 cycles, indicating that more MoO_2_ underwent conversion reaction^20^,^30^. This activation process, which has been reported many times, can be ascribed to the transformation from insertion reaction to conversion reaction, corresponding to the slowly decreasing of discharge plateaus at about 1.5 V^21^.

**Figure 8:**
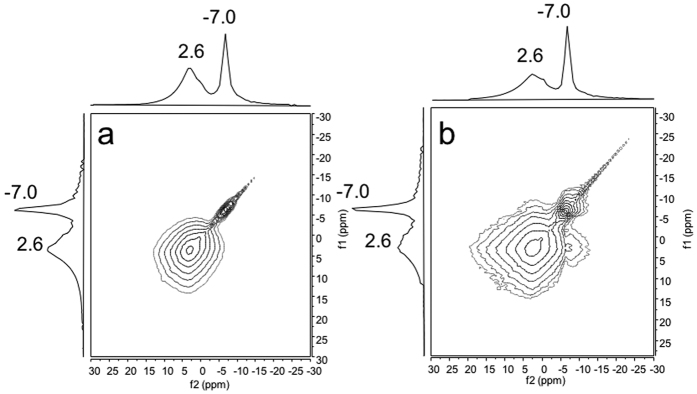
^7^Li 2D-exchange NMR spectra of MMO lithiated to 5 × 10^−3^ V with (**a**) (mixing time = 1 μs) and (**b**) (mixing time = 100 ms). Spinning rate: 20 kHz.

Two-dimensional exchange (EXSY) ^7^Li spectra provides more detailed information on the structure of MMO and additional evidences for its better electrochemical properties^43^,^44^. With a short mixing time of 1 μs, only two peaks on the diagonal at (2.6, 2.6) and (−7.0, −7.0) corresponding to Li_2_O and Li_0.98_MoO_2_ can be observed in the 2D EXSY spectrum (Fig. 8a). However, small cross peaks at (2.6, −7.0) and (−7.0, 2.6) can be observed at a much longer mixing time of 100 ms for MMO (Fig. 8b) while no peak is present at the same mixing time for MMM (Figure S3). It suggests that the two species in MMO are close in proximity and this mixing time is long enough for chemical exchange processes to occur in which lithium ions move across the interfaces between the two sites. The conclusion that the insertion and conversion reaction products Li_0.98_MoO_2_ and Li_2_O are close in space implies that MoO_2_ and MoO_3_ regions are well mixed at the microscale in MMO, while this is not the situation in the sample MMM. The much lower capacity in MMM is presumably associated with the large volume change of MoO_3_ (104% volume change) during cycling^27^,^45^ and the resulting aggregation and/or sluggish kinetics of lithium ion insertion for the MoO_2_ nanoparticles^28^,^29^. Since the MoO_2_ and MoO_3_ species are not well mixed at the microscale for MMM, inhomogeneous distribution causes phase isolation during cycling, which will lead to pulverization and rapid capacity decay. It has been demonstrated that superior stability can be achieved for the sample with a closer combination between mixed valence lithium vanadates formed in an *in*-*situ* transformation than other common coating methods^14^,^46^. The boost of the electrical performances may be explained as the results of synergistic interactions^34^,^47^,^48^,^49^,^50^. Since transformation from MoO_2_ to Li_0.98_MoO_2_ in insertion reaction only involves small volume/structure change in the very initial cycles, the well dispersed MoO_2_ helps to keep the integrity of the electrode and prevent the loss of active materials.

Although it is common that only insertion reaction occurs for bulk MoO_2_, nanostructured MoO_2_ often undergoes conversion reaction in further lithiation, presumably due to faster kinetics^30^. According to the XRD and NMR results of lithiation samples of MoO_2_ (Figure S4), the intermediate phase Li_0.98_MoO_2_ still exist after 5 cycles, which helps stabilize the structure^44^. The much more stable Li_0.98_MoO_2_ in the lithiated MMO nanoparticles may be attributed to the high concentraion of MoO_3_ around MoO_2_ in MMO. MoO_3_ is associated with a higher equilibrium potential and a lower activation barrier compared to MoO_2_, thus conversion reaction will be easier to occur during lithiation^26^. The generated amorphous Mo and Li_2_O have poor electronic conductivity and ionic conductivity, therefore slowing down the kinetics and making further lithiation of Li_0.98_MoO_2_ more difficult^26^,^30^.

## Conclusion

In conclusion, MMO is developed from the moderately oxidization reaction of molybdenum dioxide nanoparticles. The nominal composition of the material can be calculated as MoO_2.63_, according to the TGA results. MMO shows a high cyclability of 930.6 mAh·g^−1^ after 200 cycles at a current of 200 mA·g^−1^ as the anode material for lithium ion batteries. The formation of stable Li_0.98_MoO_2_ in the lithiation of MMO is confirmed with NMR and XRD on lithiated sample. 2D NMR results confirm that MoO_2_ and MoO_3_ are well mixed at the microscale. Both insertion/extraction and conversion reaction mechanisms are involved and the fraction of MoO_2_ which undergoes the former reactions is estimated to be 16%. The insertion/extraction process stabilizes the electrode material and decrease the volume change generated from conversion reaction of MoO_3_. This synergistic interaction is believed to be the key to the excellent electrochemical performances and this approach can be applied in many other transition metal oxides to provide advanced electrode materials.

## Additional Information

**How to cite this article**: Wu, D. *et al*. Mixed Molybdenum Oxides with Superior Performances as an Advanced Anode Material for Lithium-Ion Batteries. *Sci. Rep.* **7**, 44697; doi: 10.1038/srep44697 (2017).

**Publisher's note:** Springer Nature remains neutral with regard to jurisdictional claims in published maps and institutional affiliations.

## Supplementary Material

Supporting Information

## Figures and Tables

**Table 1:** Results of peak fitting for MMO in the Mo 3d region.

Peak	Position (eV)	Relative Peak Area (%)	FWHM (eV)
Mo^4+^ (3d_5/2_)	229.3	12.0	1.0
Mo^4+^ (3d_3/2_)	232.4	8.0	1.0
Mo^6+^ (3d_5/2_)	232.2	48.0	1.9
Mo^6+^ (3d_3/2_)	235.2	32.0	2.0

**Table 2:** Comparison of the capacity of MMO in this work with those of other recently reported molybdenum oxide based materials as anodes for LIBs.

Materials	Capacity (mAh g^−1^)	Current Density (A g^−1^)	Cycle number	Year	Ref.
a-MoO_2_ particles	910	0.1	50	2012	35
a-MoO_x_-C microballs	733	2	300	2013	36
F-doped a-MoO_x_	905	0.2	50	2014	37
MoS_2_/MoO_2_ nanonetworks	1025	0.2	80	2014	38
MoO_3-x_/CNT	421	0.2	100	2014	39
MoO_2_/Mo_2_C heteronanotubes	790	0.2	140	2014	41
Standing carbon-coated MoO_2_ nanosheets ongraphene	587	1	200	2015	40
MMO	930.6	0.2	200	This Work	
