# Supplementary material for: Mixed Molybdenum Oxides with Superior Performances as an Advanced Anode Material for Lithium-Ion Batteries
Source: Sci Rep. 2017 Mar 15;7:44697. doi: 10.1038/srep44697 (PMC5353649; doi:10.1038/srep44697)
Supplement: Supporting Information [file srep44697-s1.doc]

**Supporting Information for**

Mixed Molybdenum Oxides with Superior Performances as an Advanced Anode Material for Lithium-Ion Batteries

Di Wu ,^†,&^ Rui Shen ,^†,&^ Rong Yang, ^†^ Wenxu Ji, ^†^ Meng Jiang, ^‡^ Weiping Ding^†,*^ and Luming Peng ^†,*^

^†^ Key Laboratory of Mesoscopic Chemistry of MOE and Collaborative Innovation Center of Chemistry for Life Sciences, School of Chemistry and Chemical Engineering, Nanjing University, Nanjing 210023, China.

^‡^ General Motors R&D, Warren, MI 48090, USA.

Email: [luming@nju.edu.cn](mailto:luming@nju.edu.cn); [dingwp@nju.edu.cn](mailto:dingwp@nju.edu.cn)

Figure S1. Cyclic voltammograms of (a) MoO_2_ and (b) MoO_3_ nanoparticles at a scan rate of 0.2 mV·s^-1^.


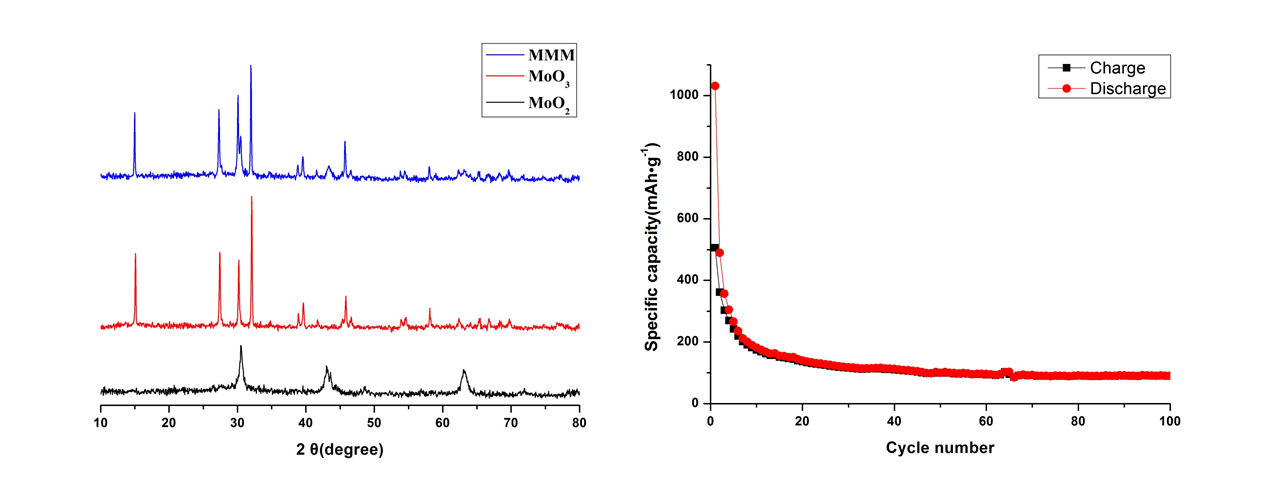


Figure S2. XRD patterns (a) and the discharge capacity as a function of the cycle number at a current density of 200 mA·g^-1^ (b) of mixed valence molybdenum oxide nanoparticles prepared by mechanical milling-mixing MoO_3_ and MoO_2_ nanoparticles (MMM, the mixture has a molar ratio of 2.63 for Mo: O). The XRD patterns of MoO_3_ and MoO_2_ nanoparticles are also shown in (a) for comparison. All of the XRD patterns shown in Supporting Information were collected by using a Bruker D8 advanced machine equipped with Co Kα radiation.


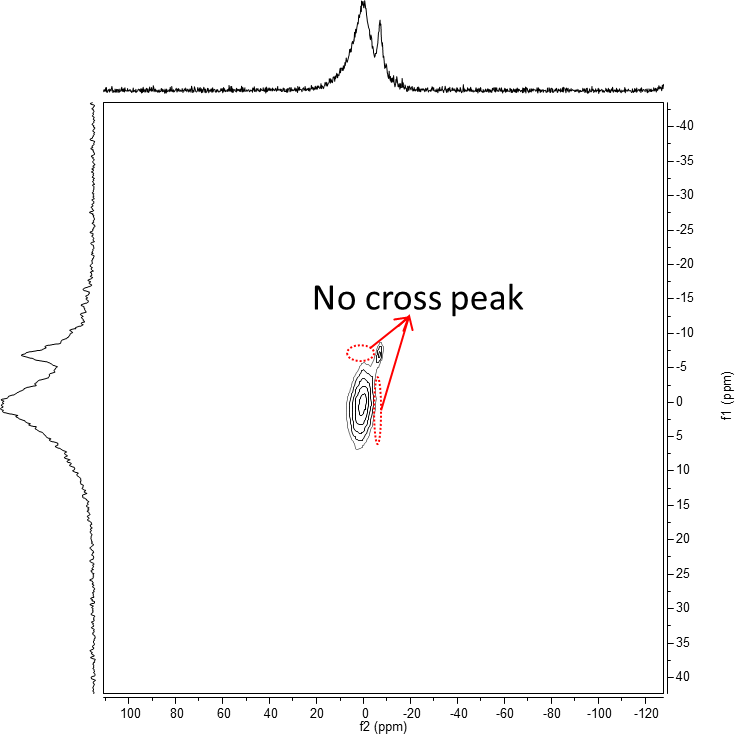


Figure S3. ^7^Li 2D-exchange NMR spectra of mixed valence molybdenum oxide nanoparticles lithiated to 0.005V (prepared by mechanical milling-mixing MoO_3_ and MoO_2_ nanoparticles) with a mixing time of 100 ms.


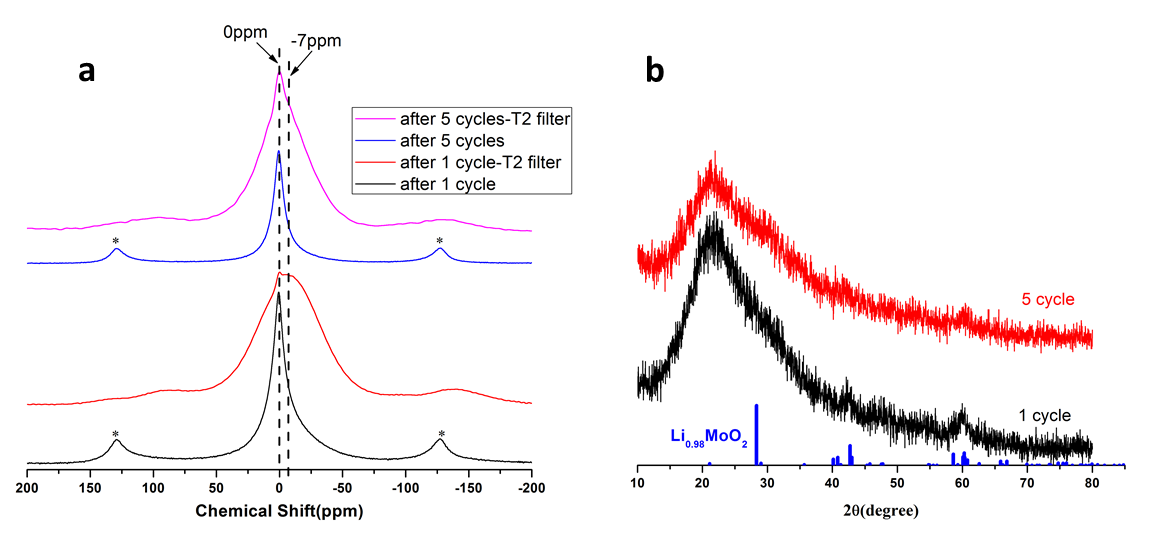


Figure S4. (a) ^7^Li spin-echo MAS NMR spectra of MoO_2_ nanoparticles lithiated to 5×10^-3^ V after 1 cycle acquired with a short τ of 55.5 μs (blue) and with a T_2_ filter (a long τ of 1110 μs, pink), in comparison to MoO_2_ nanoparticles lithiated to 5×10^-3^ V after 5 cycles acquired with a short τ of 55.5 μs (black) and with a T_2_ filter (a long τ of 1110 μs, red), spinning rate: 18 kHz; (b) XRD patterns of MoO_2_ nanoparticles lithiated to 5×10^-3^ V after 1 cycle (black) and 5 cycles (red) with the protection of a Kapton film, blue bars represent the diffraction peaks of Li_0.98_MoO_2_ (PDF#87-0392). The MoO_2_ nanoparticles are in the nanometer size regime and were annealed for a relatively short period of time. Therefore, this sample can be further lithiated, but not completely. The broad peak around 0 ppm and the broad shoulder peak at -7 ppm in the ^7^Li NMR spectra can be observed after 1 and 5 cycles. The peak centered at 0 ppm can be assigned to lithium ions in the SEI (centered at ~0 ppm) and Li_2_O (centered at 2.6 ppm) while the peak at -7 ppm can be ascribed to Li_0.98_MoO_2_, respectively. The latter is associated with a longer T_2_, and is therefore more prominent in the spin-echo NMR spectra with a T_2_-filter. The XRD pattern of MoO_2_ nanoparticles lithiated to 5×10^-3^ V after 1 cycle and 5 cycles show peaks at 2 thetas of ~43 and ~60 degree, which can be assigned to the Li_0.98_MoO_2_ phase. Both the NMR and XRD data confirm the presence of Li_0.98_MoO_2_ after 5 cycles.
